# Using questionnaires and task-related EEG signals to reveal hindered reappraisal and biased suppression in individuals with high schizotypal traits

**DOI:** 10.1038/s41598-020-62283-6

**Published:** 2020-03-26

**Authors:** Dong-ni Pan, Delhii Hoid, Zhen-hao Wang, Yi Wang, Xuebing Li

**Affiliations:** 1 Key laboratory of Mental Health, Institute of Psychology, Chinese Academy of Sciences, Beijing, 100101 China; 2 Department of Psychology, University of Chinese Academy of Sciences, Beijing, 10049 China

**Keywords:** Schizophrenia, Emotion

## Abstract

Although impaired ability to regulate emotion is commonly reported in schizophrenic patients, the exact pattern of regulation of negative emotions in high-risk individuals remains unclear. In the current study, 26 high-schizotypy individuals paired with 26 controls completed an emotion regulation questionnaire (ERQ) and a laboratory emotion regulation task with electroencephalogram (EEG) recording. Two emotion regulation strategies, namely, reappraisal and expression suppression, were concurrently examined. The late positive potential (LPP) and frontal alpha asymmetry (FAA) were selected as two independent neural indicators of the emotion regulation effect. In the ERQ questionnaire, individuals in the high schizotypy group reported higher habitual use of suppression than the controls. During the emotion regulation task, the high  schizotypy group showed no early LPP reduction in reappraisal compared with the control group and exhibited a general negative FAA pattern (left-biased alpha). In conclusion, we found that individuals with high schizotypy exhibited maladaptive regulation of negative emotions, manifested in hindered reappraisal and biased suppression; this may exacerbate the negative affect of such emotions and further serve as a risk factor for psychosis conversion. Early interventions targeting the regulation of negative emotions may be beneficial for individuals with high schizotypal traits.

## Introduction

Individuals with schizophrenia spectrum disorders have been widely recognized to show abnormalities in the processing of emotions and affective experience. One of the important aspects of this processing is that these individuals consistently report elevated negative affectivity (a stable tendency to experience negative emotions)^1^. Several researchers have suggested that this diffuse negative affect may be related to impaired emotion regulation and have demonstrated dysfunctions in emotion regulation in patients with schizophrenia^2–5^. Conceptually, emotion regulation denotes our ability “to influence which emotions we have, when we have them, and how these emotions are experienced or expressed”^6^. It refers to a sophisticated process involving *specific* and *diverse* strategy use^7^ in which reappraisal and suppression are the two strategies most widely applied^8^. Reappraisal is a cognitive-linguistic strategy intended to modulate an emotional response by reconstructing thoughts and beliefs about the meaning of a stimulus or situation. In contrast, suppression refers to the modulation of responses by suppressing behaviors associated with emotional reactions (facial expression, verbal expression, gestures, and other behaviors)^9^. Although both strategies momentarily reduce negative emotional experience, reappraisal has been generally considered more adaptive given its long-term association with personal well-being^6,10^. Suppression, on the other hand, might have detrimental effects (e.g., increased sympathetic activation) and undesirable long-term consequences for mental health^6,9^.

Using self-report questionnaires, some researchers found that individuals with schizophrenia habitually exhibit a strategy selection that is biased towards less reappraisal^2,11–13^ and more suppression^2,11,13^. Furthermore, in schizophrenia patients, a preponderance of reappraisal was found to be associated with better social functioning and greater daily positive emotion, while more frequent use of suppression resulted in greater social difficulties and increased episodes of dysphoric mood^11,14,15^, indicating the clinical significance of emotion regulation ability in schizophrenia.

Nevertheless, schizophrenia is a spectrum disorder^16^. There is substantial and increasing evidence showing that, although they fall outside the diagnostic boundaries specified in the current diagnostic systems (e.g., the ICD and the DSM), some aspects of the phenomenology of schizophrenia can also be found in the general population^17–19^. The phenomenology of “schizotypy” expresses the inter-individual continuum of schizophrenia and refers to a complex construct that is intimately related to psychotic-spectrum disorders or to stable traits that resemble the signs and symptoms of schizophrenia^16,20–22^. Individuals with high levels of schizotypal traits have been shown to display alterations in neurocognitive task performance (e.g., deficits in cognition, perception, and motor control) and altered brain structure (e.g., reductions in volume/cortical thickness in frontal and temporal areas, a lower ratio of prefrontal cortical area to temporal cortex area, and abnormalities in subcortical structures) similar to those of patients diagnosed with schizophrenia^23^. Highly schizotypal individuals may be undetected carriers of schizophrenia risk alleles^24–26^, and the existence of such a continuum may provide important clues regarding the etiology of schizophrenia^17,23,27^. Indeed, although information on deficits in emotion regulation in chronic patients with schizophrenia has been obtained^2,4^, it is not clear whether abnormal emotion regulation is associated with the onset of the disease. The defects in emotion regulation seen in schizophrenia patients may stem from the side effects of medication, since emotion regulation ability may greatly depend on cognitive functions (e.g., executive function and working memory) that are substantially affected by pharmacological treatment^28^. Reduced social contact induced by hospitalization may also lead to abnormalities in emotional characteristics^29^. Other treatments in patient studies may also affect emotion regulation. For example, a study reported that patients with schizophrenia can effectively downregulate their negative emotions, but 80% of the participants in that study had received cognitive behavior therapy^30^. Given the perspective of a schizophrenia continuum, studying individuals who exhibit high schizotypy, are free from problems stemming from the use of chronic medication or other treatments and whose responses can be measured using objective psychometric questionnaires as a model system of schizophrenia can provide a reliable and less expensive^31^ method that can be used to better elucidate the relationship between emotion regulation and schizophrenia.

Emotion regulation in high schizotypy  individuals is also an important topic for investigation in its own right. Longitudinal studies have indicated that measures of schizotypy are directly predictive of psychosis conversion^32^, suggesting high schizotypes as an obvious high-risk group^21^. Furthermore, negative emotions can trigger psychotic symptoms^33–35^, and the ability to downregulate negative affect can be crucial for those with high schizotypal traits. The capacity to effectively regulate negativity can act as a protective factor against the onset of psychosis, while the lack of such a capacity may increase vulnerability to psychiatric conversion. A previous study reported dysregulation of *positive* emotions in high schizotypy, reflecting biased habitual suppression and impaired amplification^14^; however, there may be important differences in the ability to regulate positive and negative emotions^36^. More recently, van der Meer *et al*. (2014) focused on another high-risk population, the non-affected siblings of schizophrenic patients, and found abnormal neural patterns (e.g., BOLD hypoactivation in the left frontal regions) when these individuals were regulating negative emotions through reappraisal^5^. However, at present, little is known about the pattern of negative emotion regulation in high schizotypes.

Considering that emotion regulation involves not only subjective feelings but also cognitive operations, cognitive neuroscience techniques such as electroencephalography (EEG) can be used to provide more profound and objective evidence for individual differences in emotion regulation. Two electrophysiological indicators, the late positive potential (LPP) and frontal alpha asymmetry (FAA), have been found to be strongly correlated with emotion regulation. Specifically, the LPP is a slow wave of event-related potential (ERP) that typically begins 300 ms after stimulus onset and lasts for more than one second^37^. It can be observed prominently at parietal-occipital electrodes when stimuli with high arousal and emotional significance are presented^38,39^. The LPP is of long duration and can be divided into several time windows. The early window may involve increased attention to motivation-related stimuli, while the late window indexes further affective processing (e.g., emotional elaboration); as a whole, the LPP can effectively reflect the intensity of an individual's emotional response to stimuli^38^. Decreased LPP amplitude is associated with emotion regulation strategies such as attentional shifting^40–42^, distraction^43^, reappraisal^37,44,45^, and suppression^46,47^. In addition, the LPP has been used to probe the affective processes associated with vulnerability to various psychiatric disorders^39,48^. Specifically, in schizophrenia patients and healthy controls, comparable LPP responses were evoked when the subjects passively viewed aversive pictures^49,50^, but, unlike the healthy controls, the schizophrenia patients were not able to reduce the LPP amplitude by employing reappraisal hints^2,4^.

The other indicator, FAA, refers to the difference between homologous measures of EEG alpha power at right and left frontal sites^51,52^. Because alpha power is often considered inversely related to cortical activity^53,54^, relatively larger alpha power at right electrode sites can be an indicator of relatively higher left cortical activity. The FAA typically connotes the affective motivation system: enhanced right-side alpha power (i.e., relative left cortical activation) represents positive emotions related to approach motivation (e.g., joy); conversely, enhanced left-side alpha power (i.e., relative right cortical activation) indicates negative emotions derived from avoidance motivation (e.g., fear and disgust)^55–57^. Furthermore, the FAA also characterizes emotion regulation. Enhanced right-side alpha power was shown to be positively correlated with attenuation of eye blink startle responses after subjects viewed emotion-arousing stimuli, representing the steering of emotion in a positive direction^58,59^. Reappraisal and suppression also differentially impact FAA. Habitual use of reappraisal was positively correlated with increased relative right alpha for instructed reappraisal but not with suppression^60^. In addition, studies have shown that individuals with schizophrenia generally tend to display relative left alpha power in active tasks^61^ or in the resting state^62,63^, a result that might be attributed to their generally insufficient affective motivation.

The current study aimed to investigate emotion regulation patterns in individuals with high schizotypal traits using both a questionnaire approach and a neuroscience approach. Based on previous results in schizophrenia patients, we hypothesized that a maladaptive emotion regulation pattern would also be detected in individuals with high schizotypy. Specifically, highly schizotypic individuals would exhibit reduced reappraisal ability but better use of a suppression strategy as an alternative. We hypothesized that neural indicators as well as subjective ratings would reflect this bias. For individuals with high schizotypy, LPP reduction by reappraisal may not be noticeable as in controls, but LPP reduction by suppression may be pronounced. In addition, FAA in individuals with high schizotypy would not increase (i.e., would not show relative right alpha) as in controls when reappraisal was applied.

## Results

### Demographic data

The SPQ scores of the two groups and demographic information on the participants are shown in Table 1. There were no significant differences between the two groups in gender ratio, age, years of education or fluid intelligence.

**Table 1:** Demographic information of Participants [Mean (SD)].

	Schizotypy (n = 26)	Control(n = 26)	t/χ^2^	p-value
Age (years)	20.15 (1.22)	19.57 (0.95)	1.90	0.063
Gender (male)	16	12	1.24	0.404
Education (years)	13.35 (1.35)	12.82 (0.84)	1.72	0.092
Raven test scores	71.15(11.51)	72.50(10.97)	0.520	0.668
SPQ score	45.50 (5.67)	13.58 (8.67)	15.72	<0.001***

Schizotypy = Participants with high schizotypal personality features; SPQ > 38;

Control = Participants with low schizotypal personality features; SPQ score falls the lowest 20%

The Raven test used was the short form of Raven Advanced Progressive Matrices Test^107^, with 8 points for each correct response. Participantscompleted the test online.

SPQ = Schizotypal personality questionnaire.

### Behavior results

Descriptive statistics on the behavioral data (questionnaire results and subjective rating of emotion regulation tasks) are presented in Table 2.

**Table 2:** ERQ scores and ratings on the emotion regulation task [Mean (SD)].

	Schizotypy (n = 26)	Control (n = 26)
Tasks and questionnaires		
ERQ		
Reappraisal	5.18 (0.80)	4.94 (0.66)
Suppression	4.10 (1.00)	3.26 (1.07)
Valence rating during task		
View neutral	5.48 (0.53)	5.49 (0.51)
View negative	3.20 (0.96)	3.02 (0.86)
Reappraisal	3.99 (0.98)	4.40 (0.63)
Suppression	3.35 (1.11)	3.39 (0.93)
Arousal rating during task		
View neutral	3.33 (1.63)	3.42 (1.55)
View negative	5.16 (1.73)	5.04 (1.87)
Reappraisal	4.50 (1.81)	3.70 (1.80)
Suppression	4.88 (2.03)	4.71 (1.85)

#### Emotion regulation questionnaire (ERQ)

**Figure 1:**
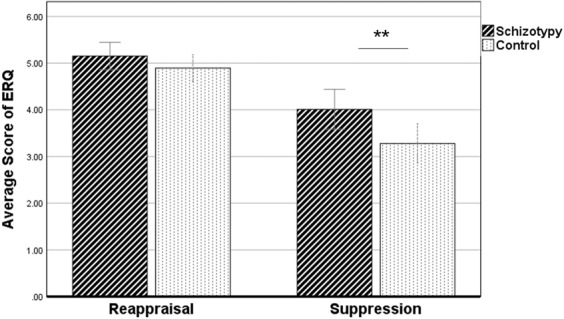
Comparison of daily strategy use between two groups. The error bars represent 95% confidence interval, **p < 0.01.

For self-reported questionnaire analyses, the total scores on the two subscales of the emotion regulation questionnaire (ERQ) were divided by the number of items in the relevant subscale to calculate the mean values of reappraisal and suppression, respectively. The independent sample t-test indicated that the use of reappraisal did not differ significantly in the high schizotypy group and the control group, *t* (50) = −1.17, *p* = 0.248; in contrast, suppression was significantly more frequently used in the high schizotypy group than in the control group, *t* (50) = −2.92, *p* = 0.005 (see Fig. 1).

#### Subjective ratings of the emotion regulation task

**Figure 2:**
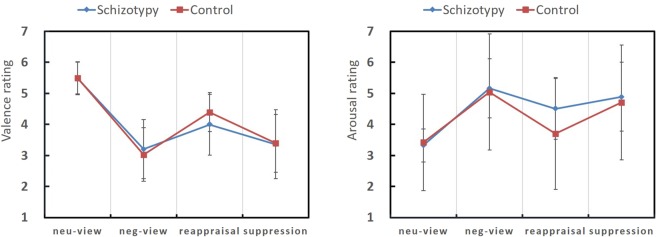
Comparison of subjective ratings (valence and arousal) in the emotional regulation tasks in the two groups. The error bars represent the 95% confidence interval.

For subjective ratings in the emotion regulation task, two-way mixed ANOVA [Group (schizotypy/control) * Condition (‘view neutral’/‘view negative’/‘reappraisal’/‘suppression’)] was conducted onemotional valence and arousal, respectively (see Fig. 2).

For the valence ratings (“higher” valence ratings indicate that the images were rated as less negative), the main effect of condition was significant, *F* (3, 150) = 108.20, *p* < 0.001, *η*_*p*_^2^ = 0.684. The valence rating of ‘view neutral’ was significantly higher than that of ‘view negative’, ‘reappraisal’ and ‘suppression’ (*p*_*s*_ < 0.001); the valence rating of ‘reappraisal’ was significantly higher than that of ‘view negative’ (*p* < 0.001), and the valence rating of ‘suppression’ was marginally higher than that of ‘view negative’ (*p* = 0.055). The main effect of group was not significant *F* (1, 50) = 0.189, *p* = 0.666, *η*_*p*_^2^ = 0.004, and the interaction between group and condition was not significant *F* (3, 150) = 1.40, *p* = 0.247, *η*_*p*_^2^ = 0.027.

To illustrate the participants’ emotion regulation *ability* more precisely, we compared the regulation effects of the two groups (*the reappraisal effect*, calculated as the valence ratings for ‘reappraisal’ minus the valence ratings for ‘view negative’, and *the suppression effect*, calculated as the valence ratings for ‘suppression’ minus the valence ratings for ‘view negative’). The independent sample t test indicated that *the reappraisal effect* of the high schizotypy group was significantly lower than that of the control group, *t* (50) = 2.21, *p* = 0.032 *(Bonferroni corrected)*, while *the suppression effect* was not significantly different in the two groups, *p* > 0.1.

Similarly, for the arousal ratings, the main effect of condition was significant, *F* (3,150) = 37.87, *p* < 0.001, *η*_*p*_^2^ = 0.431. The arousal rating of ‘view neutral’ was significantly lower than that of ‘view negative’, ‘reappraisal’ and ‘suppression’ (*p*_*s*_ < 0.01). The arousal rating of ‘reappraisal’ was significantly lower than that of ‘view negative’ (*p* < 0.001), but the difference between ‘view negative’ and ‘suppression’ did not reach significance (*p* = 0.206). The main effect of group was not significant, *F* (1, 50) = 0.314, *p* = 0.578, *η*_*p*_^2^ = 0.006; the interaction between group and condition showed a trend, *F* (3, 150) = 2.36, *p* = 0.073, *η*_*p*_^2^ = 0.045. To further explore the emotion regulation ability within each group, a group comparison of the regulation effects was conducted (*the reappraisal effect*: the arousal rating for ‘view negative’ minus the arousal ratings for ‘reappraisal’; *the suppression effect:* the arousal ratings for ‘view negative’ minus the ratings for ‘suppression’). The results showed that *the reappraisal effect* of the schizotypy group tended to be lower than that of the control group *t* (50) = 1.86, *p* = 0.069 *(Bonferroni corrected)*, while *the suppression effect* was not significantly different between groups, *p* > 0.1.

### Neural indicators

#### LPP

We quantified the LPP as the average signal amplitude across three selected electrodes (P3, PZ, P4) and evaluated the LPP in the early window (350–900) and the late window (900–1500). Two-way mixed ANOVA [Group (schizotypy/control) * Condition (‘view neutral’/‘view negative’/‘reappraisal’/‘suppression’)] was conducted. For the early LPP, the main effect of condition was significant, *F* (3, 144) = 31.43, *p* < 0.001, *η*_*p*_^2^ = 0.396. The LPP associated with ‘view neutral’ was significantly smaller than the LPP associated with ‘view negative’, ‘reappraisal’ and ‘suppression’ (*p*_s_ < 0.001); the LPP amplitude associated with ‘suppression’ was smaller than that associated with ‘view negative’ (*p* = 0.012). The main effect of group was not significant, *F* (1, 47) =  = 0.149, *p* = 0.701, *η*_*p*_^2^ = 0.003, but a significant interaction between groups and conditions was found, *F* (3, 144) = 2.81, *p* = 0.042, *η*_*p*_^2^ = 0.055. Bonferroni-corrected paired comparisons were subsequently conducted in each group to examine the specific emotion regulation effects. Both the control group and the high schizotypy group showed more positive LPP amplitude in the ‘view negative’ condition thanin the ‘view neutral’ condition (*p*_s_ < 0.001). However, the effects of the two regulation strategies differed in the two groups. In the control group, the LPP amplitude associated with ‘reappraisal’ was significantly smaller than the LPP amplitude associated with ‘view negative’ (*p* = 0.033), but there was no significant difference between ‘reappraisal’ and ‘view negative’ in the high schizotypy group (*p* = 0.793). The LPP amplitudes associated with the ‘suppression’ condition indicated that both groups successfully applied suppression strategies; they were significantly decreased compared to those associated with ‘view negative’ in both groups (*p* = 0.013 for control, *p* = 0.015 for schizotypy). In the comparison of the two strategies, no significant difference in LPP amplitude between ‘suppression’ and ‘reappraisal’ was found in the control group (*p* = 0.659). In the high schizotypy group, however, the results indicated a biased strategy use; this was reflected in a significant decrease in the LPP in ‘suppression’ compared to ‘reappraisal’ (*p* = 0.036).

**Figure 3:**
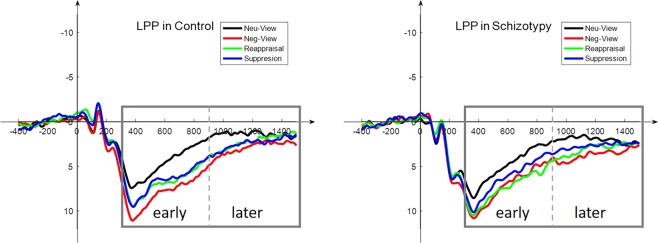
Grand average waveforms showing the LPP (mean amplitude of P3, PZ, P4) in the emotional regulation tasks in the two groups.

For the later LPP window, the main effect of condition was significant, *F* (3, 144) = 11.60, *p* < 0.001, *η*_*p*_^2^ =0.195. The LPP amplitudes observed under the ‘view neutral’ condition were significantly smaller than those observed under the ‘view negative’ (*p* < 0.001), ‘reappraisal’ (*p* =0.010) and ‘suppression’ (*p* = 0.014) conditions. The LPP amplitude observed during ‘reappraisal’ was smaller than that observed during ‘view negative’ (*p* = 0.029), while the LPP amplitude recorded during ‘suppression’ was not smaller than that observed during ‘view negative’ (*p* = 0.102). The main effect of group was not significant, *F* (1, 47) = 1.187, *p* = 0.282, and the interaction between group and conditions was not significant, *F* (3, 144) = 0.43, *p* = 0.766. The mean values of LPP amplitude are presented in Table 3, and the LPP waveforms are shown in Fig. 3.

**Table 3:** Mean LPP amplitude in the two groups and four conditions during the emotion regulation task.

group		Early LPP	Late LPP
		Mean (SD)	Mean (SD)
Control	Neu-view	4.32 (2.21)	1.23 (1.79)
	Neg-view	7.61 (2.95)	2.75 (1.56)
	Reappraisal	6.49 (1.96)	2.09 (1.87)
	Suppression	6.28 (2.45)	2.27 (1.57)
Schizotypy	Neu-view	4.53 (3.11)	1.84 (2.01)
	Neg-view	7.09 (2.80)	3.20 (2.24)
	Reappraisal	6.96 (3.01)	2.88 (2.07)
	Suppression	5.88 (2.72)	2.54 (2.12)

#### FAA

**Figure 4:**
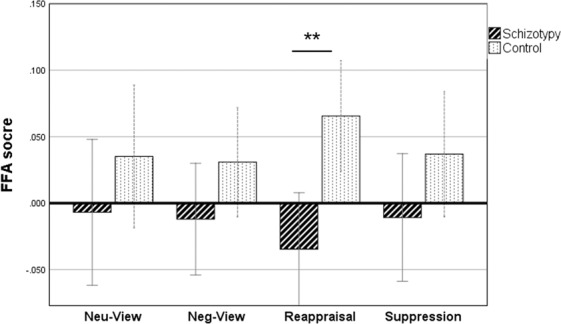
The frontal alpha asymmetry of the two groups in the emotion regulation tasks. The error bars represent the 95% confidence interval. **p < 0.05.

In the analysis of FAA scores, two-way mixed ANOVA [Group (schizotypy/control) * Condition (‘view neutral’/’view negative’/’reappraisal’/’suppression’)] was conducted. The main effect of group was significant, *F* (1,47) = 4.10, *p* = 0.049, *η*_*p*_^2^ = 0.080. The asymmetry score of the high schizotypy group was lower than that of the control group; that is, there was left lateralization of alpha (right cortical activity) in the high schizotypy group. The interaction between condition and group was marginally significant, *F* (1,47) = 2.38, *p* = 0.073, *η*_*p*_^2^ = 0.048. Post-hoc tests indicated that the alpha asymmetry score of the control group was significantly higher than that of the high schizotypy group only under the condition of ‘reappraisal’ (*p* < 0.01), revealing that left hemisphere activity during reappraisal was greater in the control group than in the high schizotypy group. No other significant main or interactive effects were observed. The results are shown in Fig. 4.

### Correlation between questionnaires, behavior data and neural indicators

Given the polarized distribution of SPQ scores as the selection criteria for the high schizotypy and control groups, we first conducted a Pearson correlation analysis separately for each group using the SPQ subscale, the ERQ subscale, subjective ratings and electrophysiological indicators during the emotion regulation task. We found a significant positive correlation between constricted affect on the SPQ and self-reported suppression of ERQ in the high schizotypy group (*r*_26_ = 0.489, *p* = 0.011). No other significant correlations were found.

Furthermore, the combined data from the high schizotypy and control groups revealed that the LPP amplitude showed a tendency to correlate negatively with valence ratings and positively with arousal ratings. The following correlations were significant or marginally significant: the early LPP was negatively correlated with the valence ratings of ‘view negative’ (*r* = −0.25, *p* = 0.051) and ‘suppression’ (*r* = −0.23, *p* = 0.075) and positively correlated with the arousal ratings of ‘suppression’ (*r* = 0.26, *p* = 0.066); the later LPP was negatively correlated with the valence ratings of ‘suppression’ (*r* = −0.24, *p* = 0.045) and positively correlated with the arousal ratings of ‘suppression’ (*r* = 0.32, *p* = 0.024).

## Discussion

Deficits in emotion regulation are well documented in schizophrenia patients^2,4^. By combining evidence from questionnaires, behavioral ratings and electrophysiological measures, the current study revealed that individuals with high schizotypy also exhibit hindered reappraisal and biased suppression.

Consistent with the findings of a previous study of non-affected siblings^5^, we here demonstrated for the first time that impaired *reappraisal* could also be detected in individuals with high schizotypal traits; evidence of this was obtained not only from subjective ratings but also from two neural indicators. Notwithstanding, the results obtained with the control group fairly replicated the results of previous studies in which LPP was decreased^38^ and FAA was increased when reappraisal was applied^60^. The absence of early LPP reduction and the absence of an increase in FAA in the high schizotypy group indicate that the highly  schizotypal  individuals were less able than normal individuals to utilize voluntary reappraisal to downregulate negative affect.

The relatively ineffective re-evaluation of threat scenarios in schizotypy may result from preconceived negative beliefs^64^ that lead to a deficiency in the ability to produce a neutral interpretation or from cognitive biases (e.g., a tendency to jump to conclusions)^65^ that impede belief updating. Indeed, researchers have demonstrated that patients with schizophrenia, particularly those with delusions, display cognitive rigidity or belief inflexibility^66^. Individuals who display high schizotypy might also be inefficient in updating their maladaptive beliefs and might ignore otherwise informative experiences, leading to the failure of cognitive reappraisal. Previous studies indicated that delusion-prone individuals are less successful in applying reappraisal^67^. Combined with our similar findings in the high-schizotypy population, failed reappraisals of threatening situations might support a state of delusional ideation^68^ and further increase the risk of psychosis conversion.

Another possible explanation for our findings might be that high schizotypes may lack the incentive to reappraise. In our study, the absence of LPP amplitude reduction in the high schizotypy group was observed in the early window but not in the later one; consistent with this, FAA tends to connote characterization of motivation rather than a regulatory effect^55–57^. This may indicate that highly schizotypal  individuals possess a relatively intact ability to process elaborate stimuli along with reluctance to invest regulatory resources proactively^38^. Studies have shown that even when provided with specific instructions on how to make neutral interpretations in negative situations, patients with schizophrenia remained unable to change their initial mental constructions^2,4,5^. Indeed, the requirement to change the established evaluation may in some way deepen the aversive response for individuals with high  schizotypy, as more left-side alpha (even compared with no regulation) was observed during reappraisal in the high  schizotypy group.

It should be noted that in this study the high schizotypy group did not report less habitual reappraisal compared to the control group on the ERQ questionnaire; this seems inconsistent with the outcome of the behavioral task. The discrepancy in the results, however, may stem from the limitations of the questionnaire method. Indeed, using ERQ alone to investigate emotion regulation in individuals within the schizophrenia spectrum can be greatly restricted by the characteristics of the participants, leading to conflicting results^2,11–13^. In addition, self-report measurements are deeply influenced by social desirability, a factor that may be more pronounced in individuals with psychosis^69^. In the current study, the behavioral task, which utilized objective neural indicators, better reflected the specific capability of reappraisal rather than the attitude toward reappraisal; thus, it offered a more sensitive method for detecting differences in reappraisal in high schizotypy individuals and controls.

Our second finding was the bias toward suppression in individuals with high schizotypy, reflected by increased frequency of self-reported suppression use; this is consistent with the results of previous studies^2,13,14^. The same effect was reflected in the laboratory-induced scenario: in the high  schizotypy group, the early LPP amplitude associated with suppression was much lower than that associated with reappraisal, suggesting highly schizotypal individuals’ preference for and excellence in using expression suppression to tone down negative emotions.

Previous studies showed that the bias toward suppression may be related to the emotional paradox in schizophrenia^70,71^ and schizotypy^72,73^ in which individuals generate strong internal feelings^49,74–77^ but exhibit flat affect or blunt external expression^14,74,78,79^. This contradictory emotional state observed in schizophrenia and in schizotypy appears to coincide with the operational processing of expression suppression, resulting in discrepancies between an individual's internal emotional state and its external expression^80^. The suppression strategy may be more natural to highly schizotypal  individuals; suppression was shown to be involved in increased sympathetic arousal in normal individuals but not in individuals with alexithymia^81,82^ or in schizophrenia patients^83^. Still, suppression is a maladaptive coping style for stress that ultimately induces a reduced but diffuse negative affect^6,9^.

It is worth mentioning that the phenomenological antagonism (poor reappraisal and excessive suppression) between the two strategies used in schizotypy and schizophrenia may not be an accidental combination. This phenomenological antagonism may represent a systematic bias indicating an overcompensation for proactive strategy impairment. Indeed, reappraisal requires implicit dynamic antecedent processes, including monitoring and anticipation of internal emotions and external situations^84^, processes that may be underdeveloped in schizotypy^85,86^. When antecedent-focused strategies (i.e., reappraisal) lose their efficacy, individuals may turn to response-focused strategies (i.e., suppression) to balance their regulatory needs^6,10^. Conversely, it is also possible that excessive use of suppression occupies limited cognitive resources and reduces the feasibility of reappraisal. The process of expression suppression was frequently reported to encroach on resources needed for other cognitive processes^87,88^, and a worsened consequence of this was low emotional comprehension and expressivity as the result of suppression^76,77^, outcomes that may in turn fuel the maladaptive emotion regulation style.

Given the schizophrenia spectrum, our study expands the affective similarity of schizotypy and schizophrenia to the domain of negative emotion regulation. This commonality may stem from an overlap in the structural and functional alterations of the brain that occur in schizotypy and schizophrenia^23^, such as abnormalities in the prefrontal cortex^22,82,89,90^, a cortical area that is vital to top-down emotion regulation processing^91^ or in the striatal and limbic regions related to dopamine release during stress-induced scenarios^92,93^. Early interventions targeting these neurobehavioral manifestations may hopefully prevent the conversion to psychotic disorders.

Although our study focused primarily on emotion regulation, we also found an absence of relative right alpha power (left cortical activity) indexed by the FAA in individuals with high schizotypy. This observed tendency is consistent with previous reports based on psychotic patients^63,94^ and, as complementary evidence, indicated a general elevated negativity in the schizophrenia spectrum. Thus, programs that offer training in regulating negative emotions may play a prominent role in intervention in schizophrenia spectrum disorders. In view of the biased suppression and hindered reappraisal ability observed in schizotypic individuals in the current study, systematic training targeting the perception and expression of emotion^95^, as well as training designed to improve the emotional flexibility of mental schema^96^, may be beneficial to these vulnerable individuals.

Several limitations of this study should be noted. First, schizotypy is a multifactorial construct that includes negative, positive and disorganized subtypes, all of which may exhibit differential emotional phenotypes^97^. Due to our small sample size and the lack of screening for positive/negative schizotypy, we were unable to examine differences in emotion regulation among individuals of different subtypes. However, one previous study^14^ reported that all three schizotypy subtypes were positively related to the daily use of suppression. In addition, a deficit in cognitive reappraisal was found both in delusion-proneness^67^, which is comparable to positive schizotypy, and in patients with chronic schizophrenia who were dominated by negative symptoms^2,4^. Therefore, a bias in emotion regulation may represent a general emotional characteristic in schizotypy. Second, the dichotomous design used in our study limits the understanding of the relationship between schizotypic traits and emotion regulation at the individual level. Our correlation analysis also failed to find a specific link between schizotypy and electrophysiological indicators of emotion regulation due to the narrow SPQ data range within groups and to other individual confounders (e.g., head circumference). Future studies are required to examine this question via correlation methods that include individuals across the entire SPQ score range rather than only artificially selected high- and low-trait groups. In addition, although comprehensive processes are involved in emotion regulation, only a limited number of strategies (reappraisal and expression suppression) were examined in this study. Other strategies, such as acceptance^98^, deserve more attention in depicting a complete pattern of emotion regulation in schizotypy. Finally, our sample, which was recruited from universities rather than from the general community, may limit the generalizability of our findings. The absence of a direct comparison of subclinical and clinical individuals does not permit demonstration of a continuous change in emotion regulation over the whole schizophrenia spectrum. Nevertheless, our study fills a knowledge gap regarding emotion regulation in schizotypy and might be informative regarding the etiology of schizophrenia and in designing methods for its early intervention.

## Methods

### Participants

Individuals with high schizotypy (the high schizotypal group) and low schizotypal features (the control group) were screened from a larger group of 704 university students in Beijing using the Chinese version of the Schizotypal Personality Questionnaire (SPQ)^20,99^. According to the scoring criteria suggested by Raine^99^, individuals who scored in the top 10% of the total sample were considered to exhibit high schizotypy. In our sample, the cut-off score was 38. The control group was randomly selected from the individuals who scored in the lowest 20% on the SPQ.

The sample size was evaluated through power analysis using G*Power 3.1.5^100^. Based on previous studies that refer to LPP comparisons of schizophrenia patients and healthy controls in the emotion regulation field^2,4^ in which the effect size f ~ 0.32, calculations indicated that at least 18 participants in each group would be required to achieve 80% power. Since the cognitive impairment associated with schizotypy is usually less than that experienced by schizophrenia patients, we aimed to appropriately increase the number of participants.

The participants volunteered to participate in the study. The following exclusion criteria were applied: a) a family history of psychosis; b) having been hospitalized in a psychiatric department; c) current or past use of antipsychotic medications; d) left- handedness. Twenty-six individuals with high schizotypy and 26 controls were included in the study. The research was approved by the ethics committee of the Institute of Psychology at the Chinese Academy of Sciences and was conducted in accordance with the approved guidelines. All participants provided written informed consent prior to the formal experiment and received a small payment as compensation.

### Emotion regulation questionnaire

The Chinese version of the Emotion Regulation Questionnaire (ERQ)^10,101^ was employed to measure the habitual use of reappraisal and suppression. This 10-item questionnaire consists of two subscales, with four items focused on suppression and six focused on reappraisal. Using a 7-point scale, participants rated to what extent the statements applied to them. Higher scores reflect frequent use of a particular strategy. The ERQ has been shown to be a reliable and effective self-report measure of emotion regulation strategies in both healthy participants^10^ and patients with schizophrenia spectrum disorders^2,5,13^.

### Emotion regulation task

One hundred and thirty-two images (560 * 420 pixels) from the International Affective Picture System (IAPS) were selected for the experiment; they included 30 neutral images associated with relatively low arousal (normative valence: M = 5.26, SD = 0.37; arousal: M = 3.23, SD = 0.53), and 102 aversive images associated with moderate arousal (normative valence: M = 2.32, SD = 0.63; arousal: M = 5.99, SD = 0.72). Of these images, 12 negative images were used for training before formal recording, and the remaining 90 negative images and 30 neutral images were used in the formal task. Specifically, the negative images were randomly divided into three groups and used in three experimental conditions: passive view, cognitive reappraisal, and expression suppression. There was no significant difference in the valence (*p* = 0.764) or arousal (*p* = 0.674) values of the three image groups.

**Figure 5:**
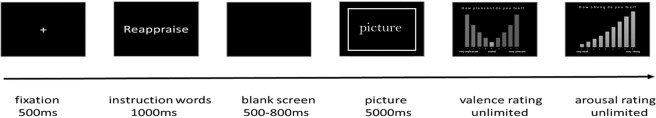
The specific trial procedure in the emotion regulation task. In the EEG task, a fixation point was initially presented for 500 ms, and then the instruction words corresponding to the block condition were presented for 1000 ms. A blank screen then appeared randomly for 500–800 ms. The picture stimulus was presented for 5000 ms. During this stage, participants were required to naturally view or apply a corresponding strategy to the block instruction. Then, participants were asked to rate their current emotional valence, from very negative to very positive (1–9), and emotional arousal, from very weak to very strong (1–9). Each block consisted of 30 trials; each participant was presented with a total of 120 trials.

Each participant was required to complete two passive attention blocks (negative-view/neutral-view) and two emotion regulation blocks (cognitive reappraisal/expression suppression). The instructions for the four blocks were adapted from previous work^88^. Specifically, the instruction for the passive view block was “Look at each picture carefully and respond to it naturally”. The instruction for the cognitive reappraisal was “Please attend to the pictures. At the same time, we want to see how you control the way you perceive these images. So, try to adopt a neutral attitude when you view them. Try to think about them objectively and analytically, not in any way that is relevant to you personally or emotionally”. The instruction for the expression suppression block was “Please attend to the pictures. At the same time, we want to see if you can control your facial expressions. Therefore, try to keep your facial muscles and do not make any expressions during picture presentation”. The order of the blocks was counterbalanced across participants. Each block consisted of 30 trials. In each trial, participants were required to view the pictures or regulate their emotions according to the preceding instructions; they then rated the valence and intensity of their emotions. The specific trial procedure is shown in Fig. 5.

### EEG recording and processing

The EEG was recorded during the task using a NeuroScan Synamp2 Amplifier. A cap containing 64 Ag-AgCl electrodes (NeuroScan Inc., Herndon, VA, USA) was placed on the scalp according to the extended International 10/20 system^102^. During EEG recording, the right mastoid was used as the reference, and signals were re-referenced to the averaged bilateral mastoids during offline data processing. All electrode impedances were maintained below 5 KΩ. EEG activity was amplified with an AC 0.05–100 Hz band-pass filter and continuously sampled at a rate of 500 Hz. EEG data were processed using EEGLAB^103^, an open source toolbox running in the MATLAB environment. EEG epochs were extracted using an analysis time window of 2000 ms (500 ms pre-stimulus and 1500 ms post-stimulus) and baseline corrected using the pre-stimulus interval. Trials contaminated by eye blinks and movements were corrected using an Independent Component Analysis (ICA) algorithm^104,105^. Trials containing activity exceeding ±100 uV were labeled as artifacts and were removed from further analysis. For each participant, at least 25 epochs remained after preprocessing for each condition. The differences in the number of remaining effective trials among both conditions and groups were not significant.

For the LPP, we selected posterior electrodes (P3, PZ, P4) for analyses according to the topographic map used in the current study and previous research experience^38^. In addition, based on previous studies^2,4^ and on the morphology of the waveforms obtained in the current study, we evaluated the LPP over the time window of 350–1500 ms, dividing it into early LPP (350–900) and late LPP (900–1500). EEG epochs were averaged across four conditions and time-locked to the onset of display of the pictures. The LPP was quantified as the average of the mean amplitude (i.e., the mean amplitude of all sampling points within the time window) of selected sites across conditions for each participant. Outliers more than 4 standard deviations from the mean were removed from analysis for each group separately. Twenty-five individuals with schizotypy and 25 controls were included in the ERP analyses (age: schizotypy: 20.14 ± 1.21, control: 19.55 ± 0.91; education: schizotypy, 13.34 ± 1.34, control: 12.81 ± 0.84; Raven test: schizotypy 75.05 ± 10.31, control: 72.00 ± 10.96; *p*_s_ > 0.1).

For FAA, Fast Fourier Transformation (FFT) with a Hanning window of 1-s width and 50% overlap was applied to all artifact-free epochs. Absolute power (microvolts-squared) in the alpha frequency band (8.5–12.5 Hz) was computed and averaged across the epochs within each condition. Our analyses focused on electrodes F4 and F3, which are the sites most commonly used in the frontal asymmetry literature^58,62,63,106^. EEG alpha asymmetry scores were calculated by subtracting natural log-transformed left hemisphere alpha power from a comparable measure derived from a homologous right hemisphere electrode (ln[F4] – ln[F3])^70^. Outliers more than 4 standard deviations from the mean were removed from analysis for each group separately. Twenty-four participants in the schizotypy group and 25 participants in the control group were included in the FAA analysis (age: schizotypy: 20.24 ± 1.00, control: 19.55 ± 0.91; education: schizotypy, 12.12 ± 1.04, control: 12.81 ± 0.84; Raven test: schizotypy: 73.12 ± 9.92, control: 72.00 ± 10.96; *p*_s_ > 0.1).

## Supplementary information

Supplementary material for emotion regulation in schizotypy.
