# Supplementary material for: Using questionnaires and task-related EEG signals to reveal hindered reappraisal and biased suppression in individuals with high schizotypal traits
Source: Sci Rep. 2020 Mar 26;10:5529. doi: 10.1038/s41598-020-62283-6 (PMC7099017; doi:10.1038/s41598-020-62283-6)

**Using questionnaires and task-related EEG signals to reveal hindered reappraisal and biased suppression in individuals with high schizotypal traits**

Dong-ni Pan^1,2^,  Delhii Hoid^1,2^, Zhen-hao Wang^1,2^, Yi Wang^1,2^, Xuebing Li^1,2,*^

^1^ Key laboratory of Mental Health, Institute of Psychology, Chinese Academy of Sciences, Beijing 100101, China

^2^ Department of Psychology, University of Chinese Academy of Sciences, Beijing 10049, China

*Corresponding author information: Xuebing Li, Key Laboratory of Mental Health, Institute of Psychology, Chinese Academy of Sciences; No 16 Lincui Rd Chaoyang District, Beijing 100101, email: [lixb@psych.ac.cn](mailto:lixb@psych.ac.cn)

**Supplementary material**

***IAPS Numbers for the Images Used in the Experiment***

Neutral Images

2038, 2102, 2214, 2393, 2480, 2580, 2620, 2745.1, 2840, 2850, 2880, 2980, 5390, 5510, 5530, 5740, 7001, 7002, 7004, 7010, 7056, 7090, 7140, 7150, 7175, 7217, 7491, 7700, 7705, 7950.

Negative Images

1050, 2095, 2141, 2683, 2710, 3001, 3016, 3059, 3068, 3103, 3168, 3195, 3500, 3550, 6022, 6212, 6313, 6520, 6563, 6830, 9050, 9253, 9410, 9419, 9470, 9490, 9520, 9600, 9622, 1201, 2703, 2880, 3005.1, 3010, 3030, 3060, 3071, 3120, 3181, 3220, 3301, 3530, 6231, 6315, 6550, 6570, 6821, 6838, 8230, 9163, 9254, 9420, 9421, 9428, 9611, 9623, 9635.1, 9901, 9910, 1930, 2799, 2900, 3015, 3051, 3064, 3101, 3130, 3185, 3225, 6021, 6250, 6312, 6510, 6560, 6571, 9075, 9250, 9400, 9412, 9414, 9425, 9430, 9433, 9491, 9911, 9920, 9921, 9940, 9941

**Figure S1. The topographic maps of LPP (early LPP 350-900ms & whole LPP 350-1500)
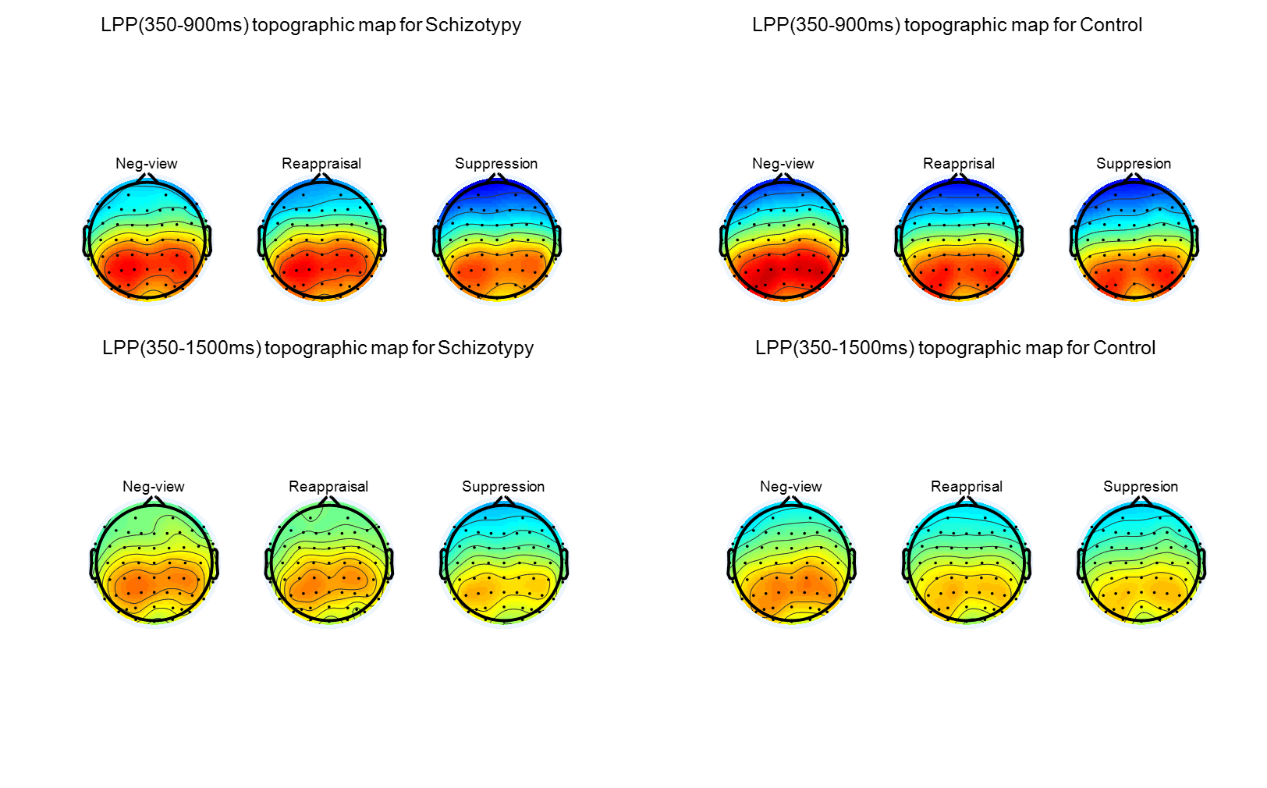
**

Figure S2


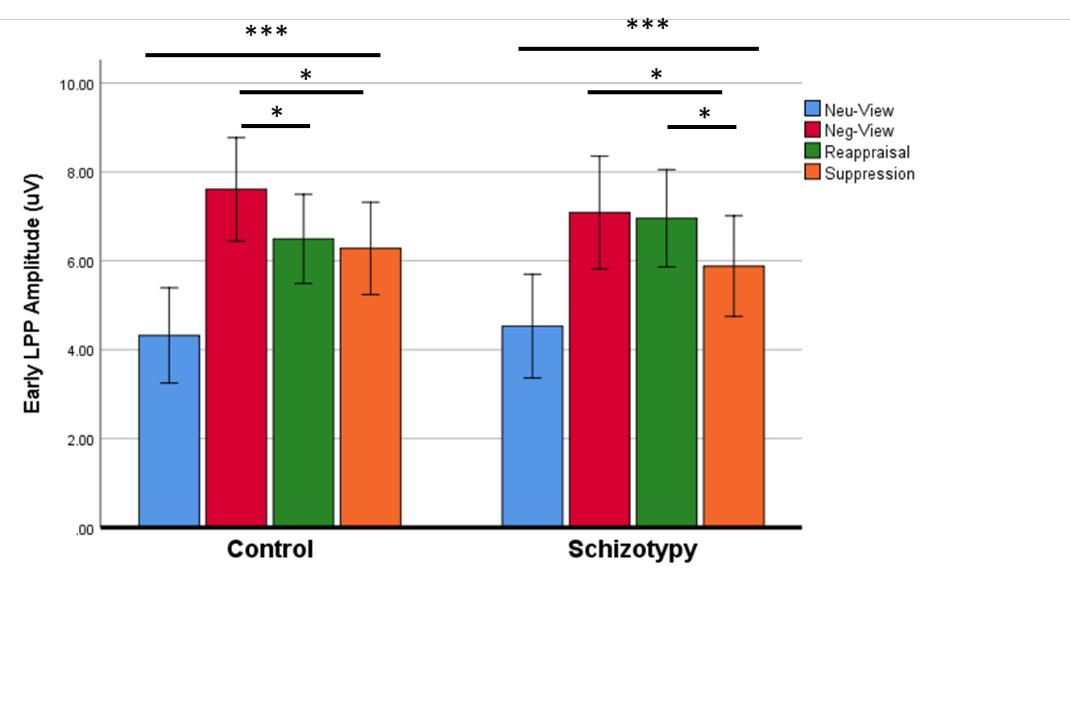

Supplement: Supplementary file 1 — Supplementary material for emotion regulation in schizotypy. [file 41598_2020_62283_MOESM1_ESM.docx]
